# Successful Endovascular Embolization of Bilateral Spontaneous Carotid Cavernous Fistula for a Patient Presenting With Bilateral Vision Deterioration: A Case Report and Literature Review

**DOI:** 10.1002/ccr3.70708

**Published:** 2025-08-01

**Authors:** Mohamed Elmarasi, Abdelrahman Elsayed, Muhammad Mohsin Khan, Mauth Hussein, Kazim Mohammed, Ali Ayyad

**Affiliations:** ^1^ Department of Medical Education Hamad Medical Corporation Doha Qatar; ^2^ Department of Neurosurgery Hamad Medical Corporation Doha Qatar; ^3^ University of Dresden Dresden Germany

**Keywords:** carotid cavernous fistula, dizziness, double vision, endovascular embolization, headache, tinnitus

## Abstract

Spontaneous bilateral CCF is rare and often misdiagnosed. Clinicians should consider CCF in patients with headache, tinnitus, or diplopia without trauma. Early imaging and treatment, such as endovascular embolization, are key to preventing serious complications and ensuring favorable outcomes.

## Graphical abstract

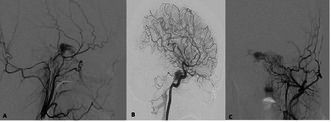

## 1 Introduction

Carotid cavernous fistula (CCF) is an abnormal communication between the carotid artery and cavernous sinus, a venous sinus located at the base of the brain [1, 2]. This leads to abnormal blood flow and elevated pressure within the cavernous sinus. CCFs can be classified based on their etiology, anatomy, and hemodynamics.

Etiologically, CCFs can be classified as traumatic and spontaneous. Traumatic CCFs are caused by direct injury to the cavernous sinus or internal carotid artery [2, 3]. They can further be classified into direct or indirect, with direct CCFs occurring when there is a rupture of the internal carotid artery within the cavernous sinus, while indirect CCFs result from communication with meningeal branches of the internal or external carotid arteries [3, 4].

Anatomically, CCFs can be classified using the Barrow classification into five types [3]. Type A CCFs involve direct communication between the internal carotid artery and cavernous sinus, while Types B and C are characterized by the involvement of meningeal branches of the external carotid artery or both internal and external carotid arteries, respectively [3, 5]. Type D CCFs are rare and result from direct communication between meningeal branches of the internal carotid artery and cavernous sinus without the involvement of the internal carotid artery [3].

In this case, we report the rare presentation of an indirect, spontaneous, bilateral type D CCF, which has not been widely documented in the literature. We also present the diagnostic imaging, management, and outcome in our patient.

## 2 Case History and Examination

**FIGURE 1:**
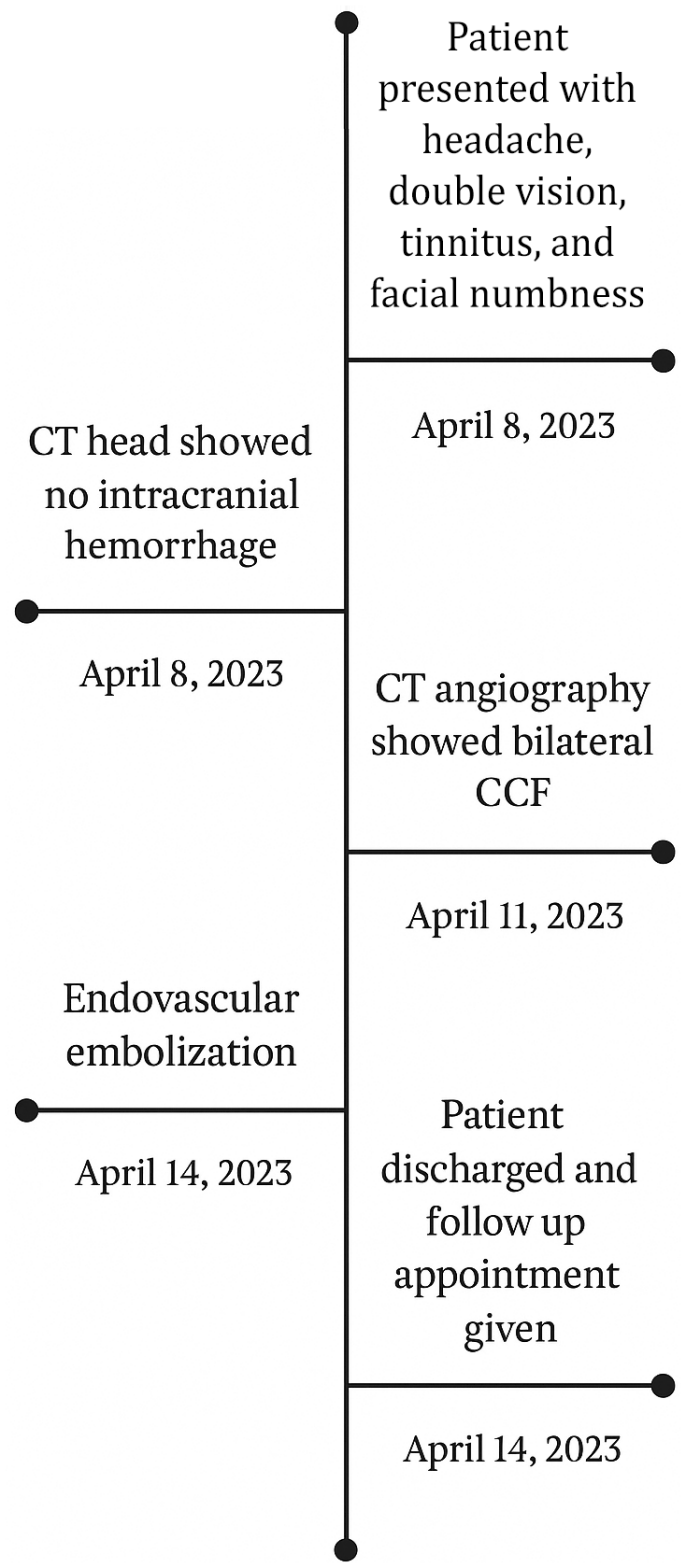
Timeline of patient admission.

A 34‐year‐old male presented to our hospital with a 1‐year history of severe headaches, dizziness, and tinnitus in the left ear, along with a 6‐month history of progressive double vision. The patient also complained of facial numbness on the left side and weakness in the left upper and lower limbs. His hospital course is summarized in figure [1]. He had no history of trauma or any significant past medical or surgical history. His family history is unremarkable. He chews tobacco but does not smoke. A summary for the timeline since the patient's admission is illustrated in Figure 1.

On physical examination, the patient had reduced visual fields on both sides, minimal right eye abduction with double vision on the right side, indicative of right abducens nerve palsy; however, no significant optic disc cupping or elevated intraocular pressures could be appreciated. Moreover, reduced sensation on the left side of the face was reported on examination. The rest of the neurological examination was normal.

## 3 Methods (Differential Diagnosis, Investigations, and Treatment)

The differential diagnosis for this patient can include a range of conditions, such as meningitis, encephalitis, subarachnoid hemorrhage, intracranial mass lesions, carotid artery dissection, and carotid cavernous fistula (CCF). In the case of our patient, the presence of intracranial hemorrhage was ruled out by CT head. Other potential causes were ruled out through a combination of physical examination, medical history review, and diagnostic imaging.

**FIGURE 2:**
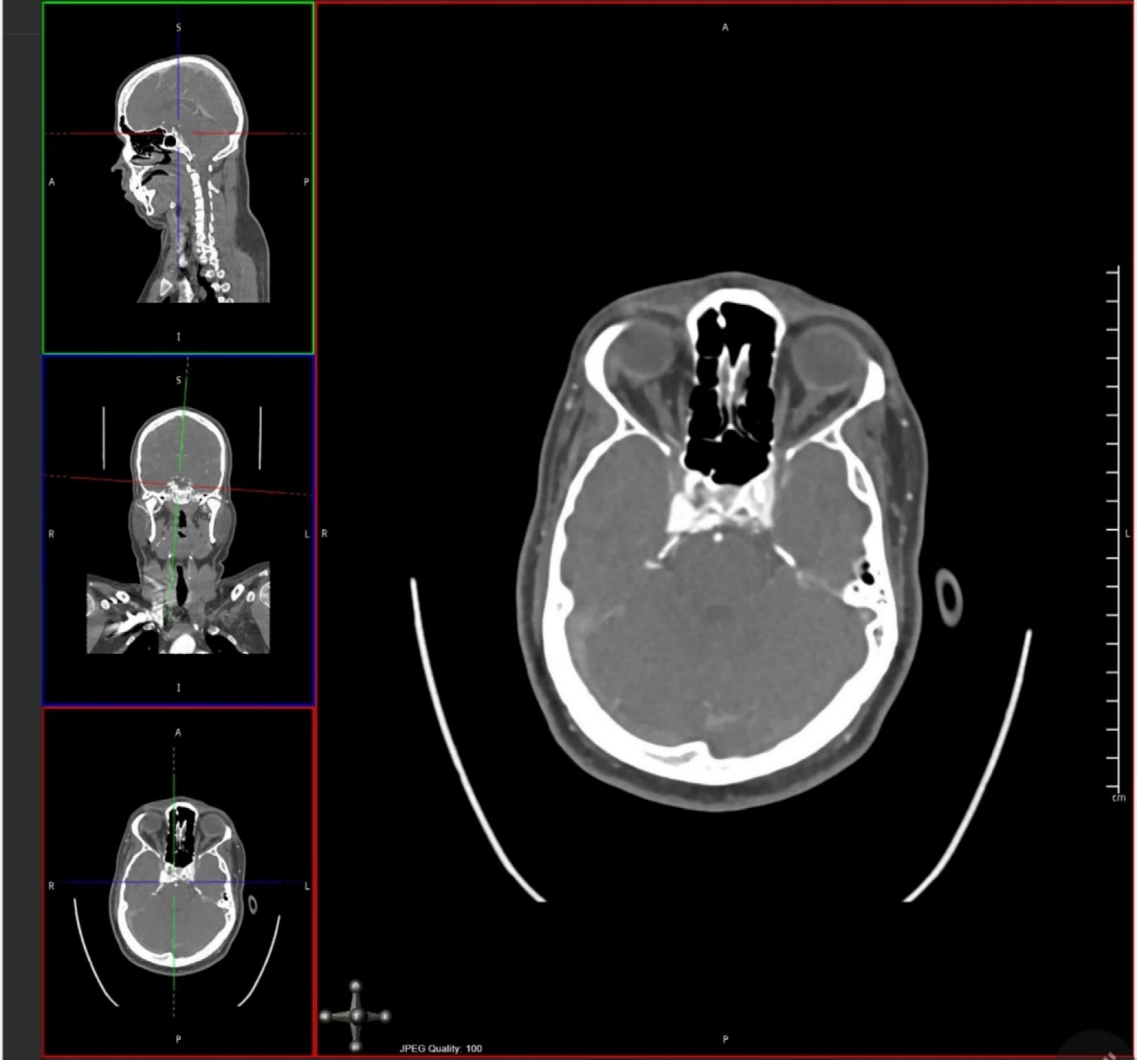
Showing multiplanar reconstructed CT images of the brain with angiographic contrast, axial, coronal, and sagittal reconstructions demonstrate markedly dilated superior ophthalmic veins and engorged cavernous sinuses bilaterally, consistent with high‐flow arteriovenous shunting.

**FIGURE 3:**
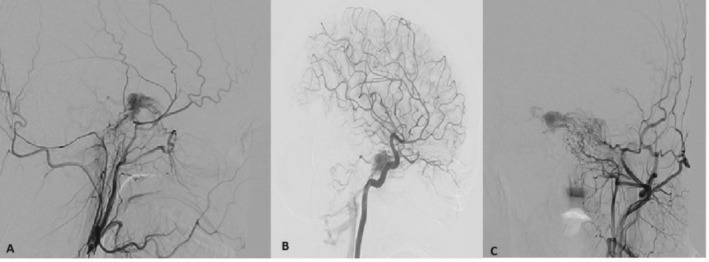
Showing cerebral angiography with External Carotid artery injection in (A,C) and internal carotid artery injection in (B): showing visualization of bilateral cavernous sinus from both external and internal carotid arteries.

CT and coronary angiography, shown in Figures 2 and 3 respectively, revealed the presence of bilateral dural carotid‐cavernous fistulas supplied by bilateral external carotid and extradural segments of internal carotid arteries, draining towards the inferior petrosal sinus without the ophthalmic vein. Digital subtraction angiography (DSA) confirmed the diagnosis of spontaneous bilateral CCFs.

## 4 Conclusion and Results

The patient was referred for endovascular intervention and underwent successful embolization of both CCFs using multiple coils. The procedure was performed under general anesthesia with endotracheal intubation. A 6F long sheath was placed in the right femoral vein, and a 5F short sheath was introduced into the left femoral artery. After that, a microcatheter was navigated to the cavernous segment of each internal carotid artery. Selective catheterization was done of the left inferior petrosal sinus and then bilateral cavernous sinuses, followed by embolization of the fistulas using multiple coils. The decision to use coils was based on their proven efficacy in achieving stable occlusion in low‐flow fistulas such as this one [6]. Left puncture side hemostasis was achieved by the angio‐seal vascular closure device, and right venous puncture side hemostasis was achieved by manual compression. This approach follows the transfemoral‐transvenous technique for catheterization and embolization of the cavernous sinus, as emphasized in previous literature [6].

**FIGURE 4:**
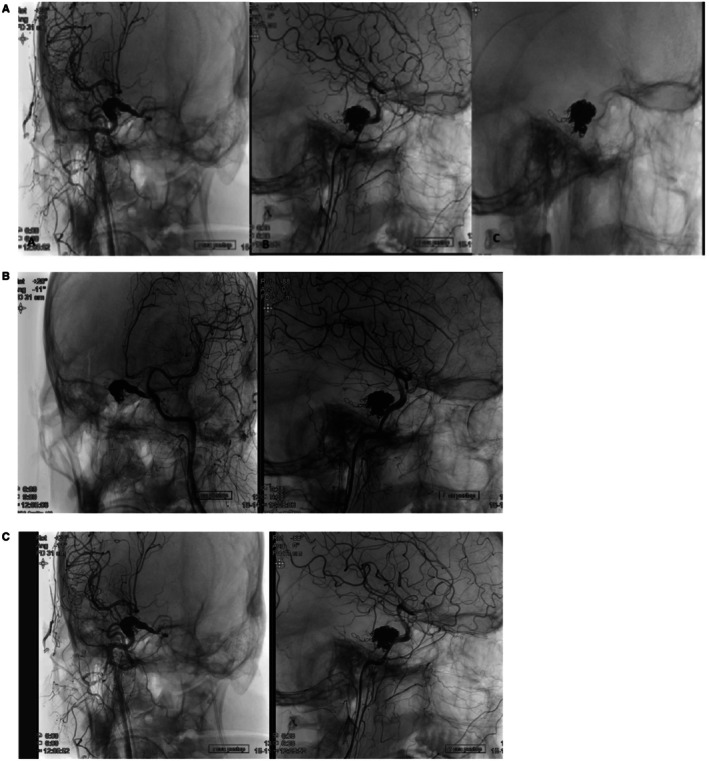
(A) Showing cerebral angiography with external carotid artery injection and internal carotid artery injection: showing complete obliteration of the fistula does not show any visualization of the sinus. (B) Left‐sided view. (C) Right‐sided view.

Post‐procedure, the patient had immediate relief of his symptoms, and repeat imaging showed complete resolution of his CCF, as shown in Figure 4. He was kept under observation for 24 h and then discharged with oral anticoagulation therapy. Follow‐up MRI and DSA at 6 months were scheduled to check for recurrent CCFs.

## 5 Discussion

In this report, we described a case of bilateral spontaneous CCF in a patient with no history of trauma and no comorbidities. Our patient was found to have an indirect, spontaneous, bilateral, Type D CCF, which is a rare form of CCF supplied by bilateral external carotid and extradural segments of internal carotid arteries in this case. Type D CCFs are often difficult to diagnose and treat due to their unique anatomy and hemodynamics. The key clinical message from this report is to share the presentation, diagnosis, and management of this rare pathology.

While Type D CCFs are already infrequent, the occurrence of bilateral, spontaneous CCFs has not been widely documented in the literature. Moreover, the absence of significant trauma or previous vascular anomalies in this patient further distinguishes this case. This report highlights the diagnostic challenges and clinical implications of identifying bilateral, spontaneous Type D CCFs and underscores the importance of considering this rare condition in patients presenting with typical CCF symptoms.

In a study of 100 patients with CCF, the most common presentations of CCF were orbital bruit (80%), proptosis (72%), chemosis (55%), abducens palsy (49%), and conjunctival injection (44%) [7]. While our patient complained of facial numbness, which could be explained by trigeminal nerve involvement in the cavernous sinus, he also had left upper and lower limb weakness. Although the exact mechanism of limb weakness in this patient is not fully clear, the vascular compromise and the disturbance of normal venous drainage in the setting of bilateral CCFs could explain the observed symptoms. Even though the exact pathophysiology remains speculative, CCF can lead to altered hemodynamics, which may affect brain regions responsible for motor function.

Treatment options for CCF include endovascular embolization, surgery, and observation, with the choice of treatment depending on the type, location, and hemodynamics of the fistula as well as the patient's symptoms and overall health [7].

In Table 1, we summarized a total of 15 reports of cases of spontaneous bilateral carotid cavernous fistula in which patients did not have predisposing factors such as Ehlers‐Danlos disease or cavernous sinus thrombosis. There were other cases reported in the literature in German, Japanese, or Polish that we did not include. The table summarized patients' presentations, treatment, and outcomes. A total of 4 cases underwent conservative management, whereas 11 patients, including ours, had endovascular embolization. Ten cases had complete occlusion of the fistulas, while 3 cases had unknown reported CCF status post‐treatment. Only 1 case was lost to follow‐up, and another one had minimal residual fistulization after treatment.

**TABLE 1:** Summary and review of reports of cases of spontaneous bilateral carotid cavernous fistula found in the scientific literature.

References	Present age, sex	Initial presentation	Treatment	Outcome
Pellegrini et al. [8]	92, F	Bilateral eye redness, lid fullness, conjunctival chemosis, ophthalmoplegia, and ptosis	Lost to follow‐up	Lost on follow‐up
Tran et al. [9]	Late 50s, M	Right‐sided retro‐orbital pain and diplopia	Endovascular embolisation	CCF resolved
Sharma et al. [10]	74, F	Diplopia and headache	Trans‐venous coil embolization of both cavernous sinuses and both superior ophthalmic veins using detachable coils	CCF resolved, with remarkable improvement of the exophthalmos, conjunctival chemosis and injection in both eyes
Belhachmi [11]	22, F	Chronic headaches and progressive bilateral exophthalmos	Endovascular embolization	CCF resolved
De Blasi et al. [12]	64, M	Mild exophthalmos, diplopia, left abducens nerve palsy, left scleral hyperemia and chemosis	Trans‐arterial embolization	CCF resolved. Complete recovery of symptoms
Baig et al. [13]	60s, M	Right eye proptosis, chemosis, hyperemia, epiphora, diplopia, and blurred vision	Conservative with spontaneous resolution	CCF resolved. Complete resolution of all symptoms
Gasparian and Chalam [14]	82, F	Sudden onset of proptosis and decreased vision	Coil embolization of the right cavernous sinus via left superior ophthalmic vein approach	CCF resolved. Visual acuity and limitation in extraocular movements significantly improved with complete resolution
Khan et al. [15]	66, F	Bilateral red eyes	Coil catheterization	CCF resolved. Symptoms were resolved
Brenna et al. [16]	70, M	Intermittent, severe headaches and left eye proptosis, associated nausea, and blurriness of the left eye, as well as vertical binocular diplopia	Craniotomy for left‐sided superior ophthalmic vein cutdown and embolization approach	Minimal residual fistulization of CCF. Decreased proptosis of the left eye, minimal ecchymosis, and no pain, but with significant ophthalmoplegia
Haugen et al. [17]	74, M	Diplopia of leftward gaze, left frontal headache, left eye chemosis, and reduced visual acuity on the left	Conservative, spontaneous resolution	Unknown CCF status. Symptoms improved
Courtheoux et al. [18]	60, F	Bilateral conjunctival injection, mild nonpulsating exophthalmos (3 mm by the Hertel exophthalmometer), chemosis, and increased intraocular pressure	Endovascular coiling. Two small Gianturco steel coils were released into the superior ophthalmic vein 1 mL of the tetradecyl sulfate was injected through USCI open‐ended guide wire	CCF resolved. Marked decrease in the right conjunctival congestion was noted, and intraocular pressure was normal
Yamamoto et al. [19]	39, F	Right exophthalmos, mild chemosis, right abducens nerve palsy, intermittent right frontal headache, and right‐side tinnitus	Detachable balloon technique for embolization	CCF resolved. Exophthalmos and chemosis resolved. Left eye blindness and left abducens nerve palsy persisted
Rwiza et al. [20]	70, F	Swelling of the right eye lid, right eye chemosis and diplopia	Conservative management	Unknown CCF status. Symptoms improved
Ashraf et al. [21]	55, M	Severe intractable headache, along with proptosis during hospital stay due to Myocardial infarction.	Conservative management	Unknown CCF status. No complications or neurologic sequela
Albert et al. [22]	46, F	8 years of right ear buzzing, progressed to bilateral for 1 year. Experienced frontal headaches that prevented her from sleeping, and progressive conjunctival injection as well as blurred vision	Surgical embolization of both fistulas	CCF resolved. Ocular and vision related symptoms improved gradually

## 6 Conclusion

Bilateral spontaneous CCF is a rare condition that can present with a variety of symptoms and poses a diagnostic challenge. Early diagnosis and prompt treatment are crucial to prevent serious complications. This report emphasizes the importance of considering spontaneous CCF in the differential diagnosis of patients presenting with signs and symptoms such as headaches, dizziness, tinnitus, and double vision, especially when traditional etiologies, such as trauma, are absent. Early recognition through imaging and a thorough clinical evaluation is essential for proper management and preventing long‐term complications. The rarity of this condition underscores the need for heightened clinical awareness and comprehensive diagnostic approaches when encountering atypical CCF presentations. Endovascular treatment with embolization is an effective treatment option in most cases, as demonstrated in this case report.

## Author Contributions

**Mohamed Elmarasi:** conceptualization, funding acquisition, investigation, methodology, project administration, supervision, writing – original draft, writing – review and editing. **Abdelrahman Elsayed:** investigation, writing – original draft, writing – review and editing. **Muhammad Mohsin Khan:** supervision, visualization, writing – review and editing. **Mauth Hussein:** validation, visualization. **Kazim Mohammed:** project administration, supervision. **Ali Ayyad:** project administration, supervision.

## Consent

Written consent was obtained from the patient, in accordance with ethical guidelines, prior to the submission of the article for publication. The patient was fully informed about the nature of the publication, its objectives, and potential risks and benefits, and they voluntarily provided their consent by signing the consent form.

## Conflicts of Interest

The authors declare no conflicts of interest.

## Data Availability

Data is available upon request.
